# Preventable fractions of cancer incidence attributable to 7-years weight gain in the Norwegian Women and Cancer (NOWAC) study

**DOI:** 10.1038/s41598-021-83027-0

**Published:** 2021-02-15

**Authors:** Marisa da Silva, Maarit A. Laaksonen, Lauren Lissner, Elisabete Weiderpass, Charlotta Rylander

**Affiliations:** 1 Department of Community Medicine, Faculty of Health Sciences, UiT The Arctic University of Norway, P.O. Box 6050 Langnes, 9037 Tromsø, Norway; 2 Centre for Big Data Research in Health, University of New South Wales, Sydney, New South Wales Australia; 3 School of Public Health and Community Medicine, Section for Epidemiology and Social Medicine (EPSO), Institute of Medicine, Sahlgrenska Academy, University of Gothenburg, Gothenburg, Sweden; 4 International Agency for Research on Cancer, World Health Organization, Lyon, France

**Keywords:** Cancer, Obesity, Epidemiology

## Abstract

There is a lack of tangible measures for directed public health action to halt the increase in weight and cancer. We estimated the fraction and preventable cases of all and major body fatness-related cancers attributable to 7-years weight gain (≥ 2 kg). We assessed validated self-reported anthropometrics from 44,114 women aged 34–49 years at the enrolment in 1991–1992 and from a second questionnaire in 1998, with follow-up through December 31, 2015. Over 18 years, 3216 body fatness-related cancers and 2041 deaths were reported. Nearly 70% of women experienced weight gain and the average weight change was 4 kg. We observed a substantial proportional impact of weight gain on pancreatic cancer with a population attributable fraction (PAF) of 41.8% (95% CI 8.1–63.1) and a high absolute impact on postmenopausal breast cancer with 4403 preventable cases (95% CI 1064–7299) and a PAF of 16.8% (95% CI 4.1–27.8), and colorectal cancer with 3857 preventable cases (95% CI 1313–5990) and a PAF of 22.6% (95% CI 7.7–35.1). Avoiding weight gain over seven years in middle adulthood could have prevented a considerable proportion of the cancer burden and thousands of cancer cases in women in Norway.

## Introduction

Obesity prevalence and cancer incidence have increased worldwide with 13 cancers defined as body fatness-related^1–3^. Most studies that have estimated the risk and burden of body fatness-related cancers have used body mass index (BMI) as a proxy for body fatness measured at one point in time^3,4^. However, weight gain tends to capture increases in fat mass more precisely than BMI and is based on at least two repeated measurements and therefore less prone to misclassification^5^. Adults tend to follow upward weight trajectories^6^ and weight gain has been shown to be independently associated with several cancers^7–10^. Weight change and cancer studies most commonly assess long-term weight change from recalled age at 18 to enrolment, with various designs in relation to sample size, exposure treatment, and follow-up duration. Few studies have assessed short-term weight gain and cancer, and there are uncertainties weather the velocity and magnitude of weight gain is associated with increased cancer risk^11^. Herein, we have assessed weight change from the enrolment to a second questionnaire seven years later.

The fraction of cancer attributable to weight gain has only been evaluated for postmenopausal breast cancer^12,13^, and no study have assessed the fraction of cancer attributable to other than long-term weight change. In a recent paper, we reported that 6–7-years weight gain of 10 kg or more was associated with increased risk of all body fatness-related cancers, postmenopausal breast cancer, endometrial, and pancreatic cancer in women in Norway^8^. To facilitate translation of these results into relevant public health measures^14^, we herein estimated the fraction of all and major body fatness-related cancers attributable to 7-years weight gain. We used a smaller sub-sample of women in Norway than in our previous publication by only including women from the first wave of enrolment, which allowed us to calculate preventable cancer cases over the follow-up period of 18 years.

## Methods

### Study population

**Figure 1:**
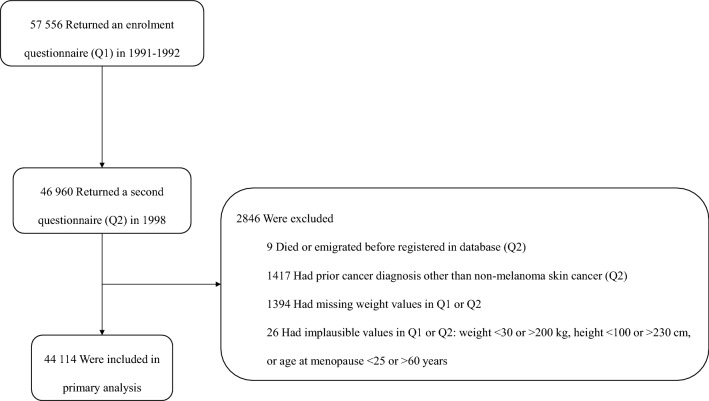
Flowchart of study participants.

The Norwegian Women and Cancer (NOWAC) study is a nationally representative prospective cohort^15^. Women were randomly sampled from the National Registry and were invited to answer consecutive questionnaires with questions on anthropometrics, sociodemographic, lifestyle, and reproductive factors. The unique personal identity number assigned to every resident in Norway allows for complete follow-up through linkages to national registries^16^. Details on the design of NOWAC have been described elsewhere^15^. In this study, we included women who returned an enrolment questionnaire in 1991–1992 and a second questionnaire in 1998. After exclusions, our final study sample consisted of 44,114 women, aged 34–49 years (Fig. 1).

### Follow-up and identification of cancer cases

Follow-up began after the second questionnaire in 1998, hence after the assessed weight change, and continued until cancer diagnosis, death, emigration, or end of study (31 December 2015), whichever occurred first. Incident, invasive, body fatness-related cancers (cancer of the breast [postmenopausal], colon-rectum, endometrium, ovary, pancreas, kidney [renal cell], gallbladder, gastric cardia, liver, oesophagus [adenocarcinoma], thyroid, multiple myeloma, and meningioma)^3^, were identified through linkage to the Cancer Registry of Norway where they were classified according to the International Classification of Diseases 10th Revision. In the analysis of all body fatness-related cancers combined, breast cancer was considered as postmenopausal if a woman reported postmenopausal status in the second questionnaire or had reached 53 years of age before or at breast cancer diagnosis. This age cut off has been used previously in NOWAC and is based on the Million Women Study convention^17,18^. Dates of death and emigration were ascertained through linkage to the Cause of Death Registry and the National Registry, respectively.

### Assessment of weight change and confounders

We used self-reported weight in kg from the enrolment and second questionnaire to calculate weight change over 7.1 (SD 0.6) years. In the analysis of the strength of the association between weight change and body fatness-related cancers, we further categorized weight gain into five groups: weight loss (< − 2 kg), stable weight (− 2 to < 2 kg), low weight gain (2 to < 5 kg), moderate weight gain (5 to < 10 kg), or high weight gain (≥ 10 kg), similar to several previous studies^12,19–21^. In the population attributable fraction (PAF) analysis, we estimated fractions of body fatness-related cancers attributable to weight gain under the scenario that women who gained weight (≥ 2 kg) had had stable weight (− 2 kg to < 2 kg).

We used directed acyclic graphs (DAGs) to identify confounding factors^22^. In all analyses, we adjusted for body weight (continuous), age (continuous), education (< 10 years/10–12 years/ > 12 years), physical activity level assessed by self-report on a scale of 1–10 and collapsed into three categories: (low, ≤ 4/moderate, 5–6/high, ≥ 7), smoking status (never/former/current), alcohol intake (≤ median/ > median g/day) from the enrolment questionnaire, and physical activity level change (increase/decrease/no change) and smoking status change (cessation/restart/no change) occurring at any time between the enrolment and second questionnaire. We additionally adjusted for age at menarche (≤ median/ > median age) in analyses on postmenopausal breast cancer, ovarian, and endometrial cancer, and menopausal status (pre-/peri-/postmenopausal/unknown) in analyses on ovarian and endometrial cancer. We considered diabetes an intermediate variable in the potential causal pathway between weight change and cancer and not a potential confounder^23^.

### Statistical analysis

Population characteristics by cancer cases and non-cancer cases were assessed using χ^2^ tests for categorical variables and one-way analysis of variance or Kruskal–Wallis test for continuous variables. We used piecewise constant hazard models to estimate hazard ratios (HR) and their 95% confidence intervals (CI) to analyse the strength of the association between weight change and all and major body fatness-related cancers^24^. Owing to the small number of incident cases, we did not perform site-specific analyses for cancers of the gallbladder, gastric cardia, liver, oesophagus, and thyroid, nor for multiple myeloma or meningioma. We fitted an age-adjusted model and a multivariable model for each outcome. We excluded women with missing information on any of the included variables, and in the site-specific analyses we excluded women who at the second questionnaire were premenopausal (postmenopausal breast cancer analysis) reported hysterectomy (endometrial cancer analysis) or reported bilateral oophorectomy (ovarian cancer analysis). In addition, we tested for interactions on the multiplicative scale between weight change and BMI status in all analyses, hormone therapy (HT) use in postmenopausal breast cancer analysis, and menopausal status in endometrial and ovarian cancer analyses. We used the likelihood ratio test to compare models with and without the interaction terms. In sensitivity analyses, we excluded the first two years as well as the first four years of follow-up to minimize potential reverse causality as weight change can be a symptom of cancer prior to clinical diagnosis.

To calculate the PAF of body fatness-related cancers attributable to 7-years weight gain, we used a recently developed method^25^ and program^26^ that accounts for death as a competing risk and statistical uncertainty. The method combines the strength of the association between weight gain and cancer, and weight gain and death, as well as the prevalence of weight gain. As the method accounts for death as a competing risk, the possibility of overestimating PAFs is reduced^25^. Further, we multiplied PAF estimates by national incidence figures from 1998 to 2015, which allowed us to estimate the number of cancer cases attributable to weight gain. All analyses were performed using STATA version 15.1 (Stata Corp., College Station, TX, USA) and SAS 9.4 (SAS Institute, Inc., Cary, NC, USA).

### Disclaimer

Where authors are identified as personnel of the International Agency for Research on Cancer/World Health Organization, the authors alone are responsible for the views expressed in this article and they do not necessarily represent the decisions, policy or views of the International Agency for Research on Cancer/World Health Organization.

### Ethics approval

This study was performed in line with the principles of the Declaration of Helsinki. Approval was granted by The Regional Committee for Medical Research Ethics in Northern Norway (P REK NORD 141/2008) and the Norwegian Data Inspectorate.

### Consent to participate

All women provided written informed consent for participation and data linkage.

### Consent to publish

All women provided written informed consent for publication.

## Results

### Exposure prevalence and population characteristics

Between the enrolment and second questionnaire, 69.3% of women gained weight (≥ 2 kg) and 24.0% had stable weight (− 2 to < 2 kg), the average weight change was 3.9 (SD 5.2) kg. Women who returned the second questionnaire did not differ considerably from women who did not, apart from being less likely to smoke (data not shown). At enrolment, the average age and BMI (SD) were 41.1 (4.3) years and 23.0 (3.3) kg/m^2^. Women with subsequent body fatness-related cancer were older, heavier, more likely to have less years of education, low physical activity, and to have experienced 7-years weight gain, compared to those without these cancers (Table 1).

**Table 1:** Population characteristics of participants according to diagnosis of body fatness-related cancer.

	Body fatness-related cancer			
	N	Cases	Non-cases	P-value
**Characteristics at the enrolment**				
Women, n	44,114	3216	40,898	
Age, mean (SD), y	44,114	42.7 (4.1)	41.0 (4.3)	< 0.001
Weight, mean (SD), kg	44,114	65.0 (10.3)	63.7 (10.0)	0.040
Height, mean (SD), cm	44,106	167.0 (5.6)	166.6 (5.5)	0.361
BMI, mean (SD), kg/m^2^	44,106	23.3 (3.4)	22.9 (3.3)	0.606
BMI, n (%)	44,106			
Underweight		88 (2.7)	1429 (3.5)	
Normal weight		2349 (73.0)	31,023 (75.9)	
Overweight		613 (19.1)	6775 (16.6)	
Obesity		166 (5.2)	1663 (4.1)	< 0.001
Education, n (%)	43,537			
< 10 years		743 (23.5)	8770 (21.7)	
10–12 years		833 (26.3)	10,017 (24.8)	
> 12 years		1592 (50.3)	21,582 (53.5)	0.002
Physical activity level, n (%)	40,278			
Low		839 (28.3)	9284 (24.9)	
Moderate		1215 (41.0)	15,710 (42.1)	
High		908 (30.7)	12,322 (33.0)	< 0.001
Smoking status, n (%)	44,114			
Never smoker		1062 (33.0)	14,060 (34.4)	
Former smoker		976 (30.4)	12,559 (30.7)	
Current smoker		1178 (36.6)	14,279 (34.9)	0.121
Alcohol intake, mean (SD), g/day	43,829	3.7 (6.5)	3.3 (5.5)	< 0.001
**Changes from the enrolment to second questionnaire**				
Weight change, n (%)	44,114			
Weight loss (< − 2 kg)		217 (6.8)	2755 (6.7)	
Stable weight (− 2 to < 2 kg)		703 (21.9)	9872 (24.1)	
Low weight gain (2 to < 5 kg)		920 (28.6)	11,684 (28.6)	
Moderate weight gain (5 to < 10 kg)		960 (29.9)	11,728 (28.7)	
High weight gain (≥ 10 kg)		416 (12.9)	4859 (11.9)	0.031
Physical activity level change, n (%)	38,295			
No change		1401 (49.7)	17,821 (50.2)	
Decrease		792 (28.1)	9582 (27.0)	
Increase		626 (22.2)	8073 (22.8)	0.442
Smoking status change, n (%)	44,114			
No change		2662 (82.8)	34 160 (83.5)	
Restart		188 (5.9)	2480 (6.1)	
Cessation		366 (11.4)	4258 (10.4)	0.211

*BMI* body mass index, *SD* standard deviation.

### Strength of associations

In total, 3216 incident body fatness-related cancers and 2041 deaths were observed over 18 years of follow-up. The average follow-up time and age at diagnosis (SD) were 16.2 (3.1) and 59.8 (5.6) years. Seven years weight gain was associated with all body fatness-related cancers, postmenopausal breast cancer, colorectal, and pancreatic cancer (Table 2). Kidney cancer was also associated with weight gain but with large confidence intervals on both sides of 1 and the analysis was hampered by few cancer cases. Further, there was not enough evidence to confirm an association between weight gain and endometrial and ovarian cancer. We did not find evidence for interactions between weight change and BMI status in any of the analyses, between weight change and HT use in the postmenopausal breast cancer analysis, or between weight change and menopausal status in the endometrial and ovarian cancer analyses. The results did not substantially change by excluding the first two or four years of follow-up, except for colorectal cancer. When excluding the first four years of follow-up, the strength of the association between weight gain and colorectal cancer considerably changed. Therefore, the first four years of follow-up are excluded in all colorectal cancer analyses presented. Further, there were no associations between weight gain and death from causes other than body fatness-related cancers.

**Table 2:** Weight change and risk of body fatness-related cancer.

	N	Cancer cases	Age-adjusted model	Multivariable model
			HR (95% CI)	HR (95% CI)
All body fatness-related cancers^a^	37,742	2775		
Weight loss (< − 2 kg)	2473	195	1.20 (1.02–1.42)	1.12 (0.95–1.32)
Stable weight (− 2 to < 2 kg)	9016	599	Reference	Reference
Low weight gain (2 to < 5 kg)	10,846	801	1.13 (1.02–1.26)	1.15 (1.03–1.27)
Moderate weight gain (5 to < 10 kg)	10,931	817	1.15 (1.04–1.28)	1.13 (1.02–1.26)
High weight gain (≥ 10 kg)	4476	363	1.30 (1.14–1.48)	1.22 (1.07–1.39)
Postmenopausal breast cancer^b^	13,074	619		
Weight loss (< − 2 kg)	892	41	1.21 (0.85–1.72)	1.20 (0.83–1.71)
Stable weight (− 2 to < 2 kg)	3118	122	Reference	Reference
Low weight gain (2 to < 5 kg)	3736	180	1.26 (1.00–1.58)	1.25 (1.00–1.58)
Moderate weight gain (5 to < 10 kg)	3750	185	1.30 (1.03–1.63)	1.26 (1.00–1.59)
High weight gain (≥ 10 kg)	1578	91	1.57 (1.20–2.06)	1.48 (1.12–1.96)
Colorectal cancer^a^	37,742	506		
Weight loss (< − 2 kg)	2473	38	1.45 (0.96–2.18)	1.40 (0.92–2.13)
Stable weight (− 2 to < 2 kg)	9016	100	Reference	Reference
Low weight gain (2 to < 5 kg)	10,846	156	1.49 (1.14–1.96)	1.50 (1.15–1.97)
Moderate weight gain (5 to < 10 kg)	10,931	153	1.42 (1.08–1.87)	1.40 (1.06–1.84)
High weight gain (≥ 10 kg)	4476	59	1.45 (1.03–2.04)	1.37 (0.96–1.94)
Endometrial cancer^c^	35,405	269		
Weight loss (< − 2 kg)	2311	23	1.42 (0.88–2.29)	1.10 (0.66–1.81)
Stable weight (− 2 to < 2 kg)	8485	61	Reference	Reference
Low weight gain (2 to < 5 kg)	10,162	58	0.80 (0.56–1.15)	0.86 (0.6–1.23)
Moderate weight gain (5 to < 10 kg)	10,276	83	1.15 (0.83–1.60)	1.09 (0.78–1.53)
High weight gain (≥ 10 kg)	4171	44	1.55 (1.05–2.29)	1.29 (0.86–1.93)
Ovarian cancer^c^	37,052	192		
Weight loss (< − 2 kg)	2429	18	1.58 (0.91–2.75)	1.43 (0.79–2.58)
Stable weight (− 2 to < 2 kg)	8850	42	Reference	Reference
Low weight gain (2 to < 5 kg)	10,634	52	1.04 (0.69–1.56)	1.04 (0.69–1.57)
Moderate weight gain (5 to < 10 kg)	10,740	61	1.22 (0.82–1.80)	1.16 (0.78–1.74)
High weight gain (≥ 10 kg)	4399	19	0.95 (0.55–1.64)	0.82 (0.46–1.48)
Pancreatic cancer^a^	37,742	90		
Weight loss (< − 2 kg)	2473	5	1.42 (0.51–3.99)	1.16 (0.41–3.30)
Stable weight (− 2 to < 2 kg)	9016	13	Reference	Reference
Low weight gain (2 to < 5 kg)	10,846	30	1.96 (1.02–3.77)	2.12 (1.10–4.08)
Moderate weight gain (5 to < 10 kg)	10,931	31	2.04 (1.07–3.90)	2.15 (1.12–4.12)
High weight gain (≥ 10 kg)	4476	11	1.86 (0.83–4.16)	1.86 (0.82–4.21)
Kidney cancer^a^	37,742	85		
Weight loss (< − 2 kg)	2473	8	1.86 (0.79–4.34)	1.40 (0.59–3.32)
Stable weight (− 2 to < 2 kg)	9016	16	Reference	Reference
Low weight gain (2 to < 5 kg)	10,846	20	1.05 (0.55–2.03)	1.13 (0.59–2.20)
Moderate weight gain (5 to < 10 kg)	10,931	27	1.42 (0.77–2.64)	1.40 (0.75–2.61)
High weight gain (≥ 10 kg)	4476	14	1.86 (0.91–3.81)	1.56 (0.75–3.25)

*HR* hazard ratio, *CI* confidence interval.

^a^The multivariable model for all body fatness-related cancers, colorectal, pancreatic and kidney cancer included the variables; weight, age, alcohol intake, education, physical activity level, physical activity level change, smoking status, smoking status change. In the colorectal cancer models the first four years of follow-up are excluded.

^b^The multivariable model for postmenopausal cancer were only in women who were postmenopausal at the second questionnaire and included the variables; weight, age, age at menarche, alcohol intake, education, physical activity level, physical activity level change, smoking status, smoking status change.

^c^The multivariable model for endometrial and ovarian cancer included the variables; weight, age, age at menarche, alcohol intake, education, menopausal status, physical activity level, physical activity level change, smoking status, smoking status change.

### Population attributable fractions and preventable cancer cases

The fraction of all body fatness-related cancers attributable to 7-years weight gain (≥ 2 kg) was 9.3% (95% CI 3.5–14.8), which is equivalent to 6859 cancer cases (95% CI 2562–10 898) (Table 3). The number of all body fatness-related cancer cases attributable to weight gain does not equal the sum of the number of specific body fatness-related cancer cases as there was not enough evidence for an association between weight gain and all site-specific cancers under study. Specifically, 41.8% of pancreatic cancer cases (95% CI 8.1–63.1) could have been prevented, should women who gained weight had had stable weight, which corresponds to 1325 cancer cases (95% CI 258–2001). Under the same scenario, 16.8% of postmenopausal breast cancer (95% CI 4.1–27.8) and 22.6% colorectal cancer (95% CI 7.7–35.1) could have been prevented, translating to 4403 (95% CI 1064–7299) and 3857 (95% CI 1313–5990) cancer cases, respectively.

**Table 3:** Population attributable fractions and absolute number of cancer cases attributable to weight gain in women in Norway from 1998 to 2015.

	Modification of weight gain (≥ 2 kg) to stable weight (− 2 kg to < 2 kg)		Data from the Cancer Registry of Norway, women aged 35–75 years, 1998–2015	
	PAF, %, (95% CI)	Attributable cancer cases^a^, (95% CI)	Total no. of cancer cases in Norway	Age-adjusted incidence rate in Norway, per 100,000 person-years
All body fatness-related cancers	9.3 (3.5 to 14.8)	6859 (2562–10,898)	73,754	376
Postmenopausal breast cancer	16.8 (4.1 to 27.8)	4403 (1064–7299)	26,211	268
Colorectal cancer	22.6 (7.7 to 35.1)	3857 (1313–5990)	17,069	87
Endometrial cancer	2.6 (− 19.1 to 20.3)	NA	NA	NA
Ovarian cancer	3.5 (− 22.9 to 24.2)	NA	NA	NA
Pancreatic cancer	41.8 (8.1 to 63.1)	1325 (258–2001)	3173	16
Kidney cancer	17.9 (− 21.2 to 44.4)	NA	NA	NA

Attributable cancer cases were only calculated for outcomes with confidence intervals not including negative values.

*PAF* population attributable fraction, *CI* confidence interval, *NA* not applicable.

^a^The number of attributable cancer cases for all body fatness-related cancers is attenuated, as not all site-specific cancers under study were associated with weight gain.

## Discussion

In this nationally representative female cohort, all body fatness-related cancers, postmenopausal breast cancer, pancreatic, and colorectal cancer were attributable to 7-years weight gain. We observed a substantial proportional impact of weight gain on pancreatic cancer and a high absolute impact on postmenopausal breast cancer expressed by the number of preventable cases. The results were independent of weight at enrolment and we did not find evidence that BMI status modified the results. Thus, keeping a stable weight may be of importance irrespective of body weight. There was not enough evidence to confirm an association between weight gain and endometrial, ovarian and kidney cancer. Therefore, the fraction of all body fatness-related cancers is attenuated, which is important to stress in dissemination of the combined estimate.

There are two fundamental assumptions from a public health perspective for calculation and interpretation of PAF estimations, the exposure should be causally related to the outcome and the exposure should be amendable to intervention^27^. Causality is imperative as we calculate a counterfactual scenario; how much of a disease burden in a population could be prevented when we hypothetically eliminate the effect of an exposure. Our result suggests that, given a causal relationship, over 40% of pancreatic cancers could have been prevented, should women who gained weight had had stable weight. The World Cancer Research Fund (WCRF) conducted a systematic literature review on pancreatic cancer and weight change, which they updated in a revised report wherein none of the included studies reported an association^28,29^. Still, WCRF suggests that weight gain is associated with pancreatic cancer, but only as an interrelated aspect with other anthropometrics of body fatness. In addition, two recent studies on weight change and pancreatic cancer also reported null associations^9,10^. There are some plausible explanations to why we found a strong association between weight gain and pancreatic cancer, contrary to that of the literature. We assessed 7-years weight gain to capture rapid accumulation of weight gain, whereas most previous studies have assessed weight change from age 18 to study baseline with a weight follow-up ranging from ~ 20 to 50 years within each study. The velocity of accumulated weight gain may have unknown biological implications for cancer development^11^. More specifically, pancreatic cancer development can be related to increased insulin levels and higher bioavailability of insulin-like growth factor^30^, in which short-term weight gain, rather than long-term weight gain, may play a vital role. However, we have found no mechanistic studies that assess short- or long-term weight gain and cancer to confirm or reject this hypothesis. Moreover, in our study, all participants had potentially 18 years of follow-up. A long follow-up time is particularly important when assessing time-to-event data of pancreatic cancer, which has one of the highest median age at diagnosis (72 years in women in Norway)^31^. A study with short follow-up time and a large proportion of young participants will include person-time from individuals in a scenario where it is unlikely for them to have the time to develop pancreatic cancer. Despite relatively few pancreatic cancer cases in our study sample, which limited the precision of the estimates, our result suggest that stable weight can have a large potential for primary prevention of pancreatic cancer. If confirmed in future studies, this result may be of special importance due to the poor prognosis of the disease and given that pancreatic cancer incidence has steadily increased for decades in women in Norway and the US^31,32^.

In our study, postmenopausal breast cancer had a large number of cancers cases attributable to 7-years weight gain, which was expected as it is the most commonly diagnosed cancer in women^2,31^ and the only cancer for which WCRF declare a positive association with weight gain^33^. Further, postmenopausal breast cancer is the only site-specific cancer with reported PAF estimates attributable to weight gain^12,13^. The latest prospective cohort study that calculated fractions of postmenopausal breast cancer attributable to weight gain reported a PAF similar to our result, with comparable strength of association between weight gain and postmenopausal breast cancer, but a higher prevalence of weight gain^13^.

Colorectal cancer also had a large number of cancer cases attributable to 7-years weight gain. Although we excluded the first four years of follow-up to minimize potential reverse causality, we cannot fully rule out that the lower effect estimates in higher weight categories, was due to weight loss as a preclinical symptom of colorectal cancer and resulted in few colorectal cancer cases in these categories. There are uncertainties of the magnitude and period in which unintentional weight loss occurs before colorectal cancer diagnosis, particularly since colorectal cancer can develop over more than 10 years^34,35^. Women in Norway have the highest colon cancer incidence rates in the world, which cannot be explained by established risk factors^2^. Thus, more colorectal cancer studies are warranted, both to disentangle the effect of reverse causality and to elucidate plausible biological mechanisms of weight gain.

### Strengths and limitations

The main strength of our study is its large, nationally representative sample of women. The external validity of NOWAC is considered high, as the distribution of exposures is independent of the response rate, and the cumulative incidence of cancer is not substantially different from national figures^36^. Moreover, the comprehensive questionnaires enabled us to control for important confounders, and our study design with its long prospective follow-up is critical when investigating body fatness-related cancers that develop later in life. The method that we used accounted for death as a potential competing risk, which may be present in studies with long follow-up and aged participants^37^. Failure to account for death as a competing risk may result in overestimated risk estimates. We estimated 7-years weight change from the enrolment to a second questionnaire, which is different from most studies that have calculated weight change from recalled weight at age 18 to weight at enrolment, which may be prone to recall bias and misclassification as older women would have had a longer period of possible weight gain. Further, we have reported the fraction of body fatness-related cancer burden attributable to weight gain, which is an estimate of the preventable proportion, given a hypothetical intervention. Although the mathematics to calculate this proportion is sound, the intervention must be achievable in the target population for the estimate to fit public health action. In our study, the intervention would be to avoid weight gain over 7-years during middle adulthood, which seems less challenging than weight maintenance from young adulthood through several decades, as in many other weight change studies.

Nevertheless, this study has limitations. Weight was self-reported, and the well-established tendency to underestimate weight that increases with age and BMI, has also been confirmed in NOWAC^38,39^. However, we assume that the potential misclassification was non-differential between women with and without body fatness-related cancers. We also assume that the potential underestimation of weight was similar at the enrolment and the second questionnaire, and that the change in weight was less prone to misclassification. Although, we have adjusted for many important confounders, residual confounding may be present as we could not adjust for medical conditions that affect both weight gain and cancer, time of initiation and frequency for several confounders, nutritional aspects, as the food frequency questionnaire was only provided to a subsample of women, hereditary predisposition, or additional environmental factors that could be related to both weight gain and cancer. Pancreatic and kidney cancer, which were the least commonly reported cancers among the body fatness-related cancers under study, had relatively few cancer cases that led to large confidence intervals. Further, the precision in all PAF analyses was low with large confidence intervals. However, many PAF studies fail in reporting confidence intervals and thus the precision of the estimates is difficult to compare. Consequently, due to the large confidence intervals, the numbers of attributable cancer cases are approximations. The generalisability of our study is limited to Norway. The strength of association between weight gain and cancer may not substantially differ across regions but women in our study sample were slimmer than in many other high-income countries at that time^1^, and thus the exposure prevalence of weight gain and the PAF estimates are likely to be lower in our study.

## Conclusions

Seven-years weight gain in women in middle adulthood had an impact on the burden of all body fatness-related cancers, postmenopausal breast cancer, colorectal and pancreatic cancer. Given a causal relationship, over 40% of diagnosed pancreatic cancer could have been prevented should women who gained weight had had stable weight. Our study implicates that a substantial proportion of major body fatness-related cancers could have been prevented through weight maintenance and suggests a possible role of the velocity of accumulated weight gain in assessing cancer risk.

## Data Availability

The data underlying this article will be shared on reasonable request to the person responsible for the NOWAC study https://uit.no/research/nowac.

## Code availability

The codes used in this article will be shared upon request to the corresponding author.
